# The Association Between Number of Chronic Conditions and Benzodiazepine Prescribing in Region Stockholm, Sweden: A Total Population‐Based Cohort Study

**DOI:** 10.1002/hsr2.72970

**Published:** 2026-08-09

**Authors:** Caroline Kappelin, Caroline Wachtler, Gunnar Ljunggren, Axel C. Carlsson

**Affiliations:** ^1^ Department of Neurobiology, Care Sciences and Society (NVS) Division of Family Medicine and Primary Care, Karolinska Institute Huddinge Sweden; ^2^ Academic Primary Health Care Centre, Region Stockholm Stockholm Sweden

**Keywords:** benzodiazepines, cohort study, multimorbidity, potentially inappropriate prescribing

## Abstract

**Background and Aims:**

The aim of this study was to examine the association between an increasing number of chronic conditions and prescribing of benzodiazepines.

**Methods:**

Conditional logistic regression was used to study the association between the number of chronic conditions accumulated during a 4‐year period and risk of having ≥ 2 collected prescriptions of benzodiazepines during the 2 following years in two cohorts before and during the COVID‐19 pandemic. Data were collected from the VAL databases for the total adult population in Stockholm, Sweden between January 1, 2014 and December 31, 2019, and January 1, 2016 and December 31, 2021.

**Results:**

Of the total population with approximately 1.3 million individuals in each cohort, 3.8% of the women and 2.2% of the men had ≥ 2 collected prescriptions of benzodiazepines before the COVID‐19 pandemic. During the COVID‐19 pandemic, 3.1% of the women and 1.8% of the men had ≥ 2 collected prescriptions of benzodiazepines. The risk of having ≥ 2 collected prescriptions of benzodiazepines significantly increased in individuals with two chronic conditions, compared to the reference group, OR 3.25 (3.25–3.25) for women, and OR 3.57 (CI 3.57–3.58) for men before COVID‐19. The risk increased with an increasing number of chronic conditions, OR 11.41 (CI 11.40–11.42) for women and OR 13.26 (CI 13.25–13.28) for men with 5–9 conditions before COVID‐19. Results were similar during the COVID‐19 pandemic. The association regarded both ongoing and new prescriptions.

**Conclusion:**

In this study, individuals with an increasing number of chronic conditions were more likely to receive both ongoing and newly initiated benzodiazepine prescriptions. While the appropriateness of prescribing was not assessed in this study, these findings can inform healthcare providers and policymakers about the need for strategies to reduce such prescribing in this population.

## 1 Introduction

People with multiple long‐term conditions (MLTC), increasing with an aging population, are at risk of poor health outcomes and treatment burden. MLTC, also described as multimorbidity, are defined as having two or more chronic conditions [1]. People with MLTC are at increased risk of poor quality of life [2] and common mental health problems such as depression [3] and anxiety [4]. Yet common mental health problems are poorly diagnosed in people with MLTC [5, 6]. In addition, an increasing number of chronic conditions are associated with poorer health outcomes and increased complexity in people with MLTC [7, 8].

People with MLTC are also at increased risk of treatment burden, many visits to different healthcare providers, and polypharmacy [9]. Polypharmacy is commonly defined as having ≥ 5 prescriptions [10], and is associated with increased risks of drug–drug interactions, adverse drug reactions, and potentially inappropriate prescribing [11, 12].

Potentially inappropriate prescribing is when prescribing does not follow current medical guidelines [13] and leads to higher risks than benefits for the individual [14, 15]. Medication can be potentially inappropriate if it has higher risks than benefits for an individual regardless of dose or period of intake of the medication [16], or if medication is prescribed for longer time periods or in higher doses than recommended [17]. One example of potentially inappropriate medications is benzodiazepines [18, 19]. Benzodiazepines have been used in short‐term treatment of insomnia and anxiety, but are no longer recommended in any dose or for any time period in Sweden since 2016 [20]. This is due to lack of treatment effect over time [21], and risks of adverse drug reactions involving addiction [21], falling [22], and cognitive impairment [23] when used long‐term. Nevertheless, there are a few exceptions when benzodiazepines still have an indication such as in individuals with epilepsy [24] and for shorter periods of severe behavioral and psychological symptoms in dementia [25].

Studies are lacking describing the association between an increasing number of chronic conditions and the risk of prescribing benzodiazepines. A French national cohort study published in 2022 showed an association between an increasing number of chronic conditions and the risk of having prescriptions of benzodiazepines and Z‐drugs together, but did not analyze benzodiazepines and Z‐drugs separately [26]. In a population‐based cross‐sectional study, 22% of the population in Region Stockholm had MLTC and 10%–27% had a prescription of anxiolytics, including benzodiazepines [11]. However, there was no investigation of the association between the number of chronic conditions and prescriptions of anxiolytics [11].

The World Health Organization (WHO) has highlighted a need to improve care delivery for people with MLTC by addressing common mental health problems [27] and decreasing the harms and risks of polypharmacy, including potentially inappropriate prescribing [10]. In this study, we hypothesize that there is a positive association between an increasing number of chronic conditions and both ongoing and new benzodiazepine prescriptions.

The aim of this study was to examine the association between an increasing number of chronic conditions and prescribing of benzodiazepines.

## 2 Methods

We used the STROBE checklist for reporting cohort studies [28].

### 2.1 Study Design

We used a total population cohort study design to examine the association between the exposure measurement, having MLTC with an increasing number of chronic conditions during a 4‐year exposure period, and the outcome measurement, having ≥ 2 collected prescriptions of benzodiazepines during a 2‐year follow‐up period in the total adult population in Region Stockholm, Sweden. We chose the primary outcome to avoid including participants with a single prescription of benzodiazepines which could be appropriate in exceptional cases, such as prior to surgery. We had no data about indication or duration of prescriptions of benzodiazepines as we only had access to Anatomical Therapeutic Chemical (ATC) codes. We could have chosen to use a cut‐off value of defined daily doses (DDD) as our primary outcome. However, as benzodiazepines are considered being inappropriate regardless of dose in a Swedish context, with a few exceptions, we believed that DDD was not fruitful in our aim to address prescriptions of benzodiazepines in the population of Region Stockholm. In another country with other recommendations, a cut‐off value of DDD might have been more appropriate to use.

The association was examined in two cohorts, before and during the COVID‐19 pandemic. When planning the study, we decided to examine the association in these different cohorts because the latest available data when planning the study was from the period during the COVID‐19 pandemic. During the COVID‐19 pandemic, there were more visits to hospitals, increased risk of isolation, and poorer mental health with increased associated risk for benzodiazepine treatment [29]. Yet it was also a period of less overall contacts with the healthcare system.

Because of differences in health outcomes and healthcare utilization during the pandemic, we analyzed data during the period before the pandemic as well. We did not statistically compare results from the two time periods.

The study design did not address incidence of prescriptions of benzodiazepines. However, as we were interested in if prescribing of benzodiazepines was ongoing or new, a washout analysis was conducted as a subgroup analysis to assess a potential time correlation regarding prescribing.

### 2.2 Eligibility Criteria

All individuals aged ≥ 18 years at the start of exposure listed at a primary care practice in Region Stockholm were included. Individuals who moved in or out of Region Stockholm or died during the periods of exposure and outcome measurements were excluded.

### 2.3 Variables and Data Collection

**Figure 1:**
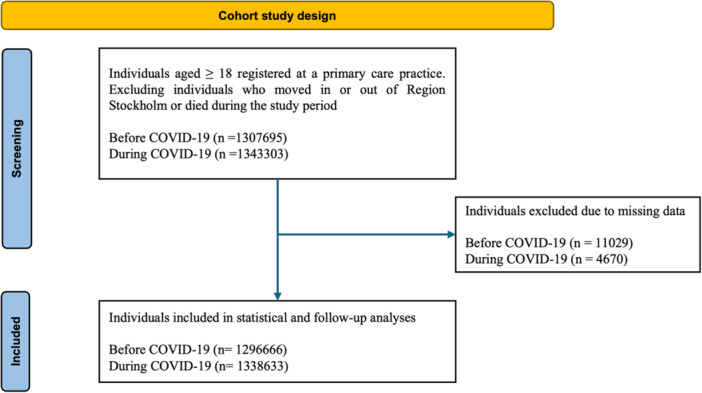
Flow chart representing the included residents in Stockholm County from January 1, 2014, until December 31, 2021.

We collected data from the VAL database, an administrative healthcare register of all citizens living in Region Stockholm, Sweden from January 1, 2014, until December 31, 2021. First, we excluded all individuals who moved in or out from Stockholm or died during the period according to eligibility criteria, as we needed continuous access to their healthcare data during the study period (Figure 1).

The outcome variable was dichotomous involving 0–1 or ≥ 2 collected prescriptions of benzodiazepines (N05BA and N05CD) from all pharmacies in Sweden. Data on outcome measurement were collected during a 2‐year period for each cohort, between January 1, 2018 and December 31, 2019, before COVID‐19, and between January 1, 2020 and December 31, 2021, during COVID‐19. Because prescriptions are valid for 12 months in Sweden, the outcome was measured over 2 years to capture the patients' benzodiazepine use.

The exposure variable was categoric involving: 0–1 (referent), 2, 3, 4, 5–9, and ≥ 10 registered chronic conditions collected during the 4 years prior to the outcome measurement, between January 1, 2014 and December 31, 2017, before COVID‐19, and between January 1, 2016 and December 31, 2019, during COVID‐19. Since many chronic diagnoses are not registered annually in Sweden, we chose 4 years of exposure to capture all relevant diagnoses [30]. Exposure data were collected using a pre‐existing definition of chronic conditions (Supporting Information S2: Table 1). This definition was developed by Barnett et al. in 2012 [31] and later adjusted to Swedish conditions, primarily by adding diagnoses of pain instead of defining chronic pain by occurrence of prescriptions of pain medication [11]. In this study, we chose to use two chronic conditions as it is the definition of multimorbidity and MLTC [7, 27]. The choice of three chronic conditions was used because ≥ 3 chronic conditions from three different organ systems is the common definition of more complex forms of MLTC [32], proposed to be more clinically relevant to capture the sicker patients than with the definition of MLTC alone [32, 33]. The choice of four chronic conditions was used as it is a more rare definition of complex forms of MLTC [34]. The choice of the two groups: 5–9 chronic conditions and ≥ 10 chronic conditions were made because ≥ 5 and ≥ 10 chronic conditions are considered to be clinically relevant cut‐off values for more severe forms of MLTC [31].

Baseline data were collected for age as a continuous variable in years and socioeconomic status as a categoric variable using Mosaic areas. Mosaic areas represent if individuals reside in neighborhoods with high (1), medium (2), and low (3) socioeconomic status [35, 36]. In addition, data were gathered on prescribing unit of benzodiazepines during the time of outcome measurement, defined as primary care, secondary care, or both. Data were compiled on potentially confounders involving: number of healthcare contacts in primary and secondary care, including hospital admissions being a continuous variable; Presence of ≥ 1 psychiatric diagnosis from ICD‐10 Chapter F being a dichotomous variable; prescence of polypharmacy, using the definition of ≥ 5 collected prescriptions of medications [10] being a dichotomous variable, reported with ATC codes. Baseline data and data on potential confounders were collected during the year before data collection of outcome measurement between January 1, 2017, and December 31, 2017, before COVID‐19, and between January 1, 2019, and December 31, 2019, during COVID‐19. Data collection of outcome, exposure, baseline, and confounder measurements was made at one point in time for each variable.

### 2.4 Statistical Analysis

Descriptive statistical analyses were made on baseline data. Baseline data were presented for individuals with or without the outcome measurement stratified by sex in one table for each cohort. Data were presented using proportions and mean values with standard deviation. Statistical analyses were made using a logistic regression model [37] calculating odds ratios (OR) with 99% confidential intervals for the outcome measurement in the two cohorts. The logistic regression model was adjusted for age and socioeconomic status and stratified by sex. The results were stratified for men and women rather than including sex in the logistic regression model, because sex differences are better visualized with stratification [38]. In additional logistic regression models, the original model was adjusted for each of the three potential confounders one by one. In the washout analysis all individuals with collected prescriptions of benzodiazepine (N05BA and N05CD) during the 2 years before outcome measurement were excluded. All statistical analyses conducted in this study were prespecified. SAS was used for statistical analyses, version 9.4 (2016, SAS Institute Inc., Cary, NC, USA).

### 2.5 Ethical Approval

This study was performed in line with the principles of the Declaration of Helsinki. The study was approved by the Swedish Ethics Committee (Registration Number 2021‐01016). All data were presented anonymously on a group level. Individual consent was not applicable due to registry‐based data.

## 3 Results

### 3.1 Individuals With Potentially Inappropriate Benzodiazepine Prescribing

Of the total population of 1,307,695 individuals before COVID‐19 pandemic, 25,885 women (3.8%) and 13,848 men (2.2%) had ≥ 2 collected benzodiazepine prescriptions. On a group level, these individuals had a higher mean age, mean number of chronic conditions, mean number of visits in primary and secondary care, as well as more hospital admissions. Moreover, they were more likely to have one or more psychiatric disease(s) and polypharmacy. In areas with low socioeconomic status, there was a slightly higher proportion of both men and women who had ≥ 2 collected benzodiazepine prescriptions. No other differences were seen between the groups regarding socioeconomic status for men or women. Benzodiazepines were prescribed in primary care for 55.9% of women and 57.3% of men, from secondary care for 28.2% of women and 26.3% of men. The rest were prescribed in both care settings (Table 1).

**Table 1:** Baseline data of individuals with and without ≥ 2 collected prescriptions of benzodiazepines before the COVID‐19 pandemic.

	Women			Men		
	≥ 2 collected prescriptions of benzodiazepines			≥ 2 collected prescriptions of benzodiazepines		
	Yes	No	Total	Yes	No	Total
Total number (*N*) and percent of patients with or without ≥ 2 collected prescriptions of benzodiazepines	*N* = 25,885 3.8%	*N* = 651,767 96.2%	*N* = 677,652 100%	*N* = 13,848 2.2%	*N* = 616,195 97.8%	*N* = 630,043 100%
Number of chronic diagnoses (mean number (*N*) and (standard deviation (SD)))	2.42 (SD 1.80)	0.96 (SD 1.27)	1.02 (SD 1.33)	2.32 (SD 1.94)	0.85 (SD 1.31)	0.89 (SD 1.35)
Number of chronic diagnoses	
0–1 (ref)	34.7%	74.8%	73.3%	39.8%	78.0%	77.2%
2	23.3%	13.6%	13.9%	21.2%	11.0%	11.2%
3	17.8%	6.5%	7.0%	15.4%	5.7%	5.9%
4	11.6%	2.9%	3.3%	10.5%	2.9%	3.1%
5–9	12.5%	2.1%	2.5%	12.9%	2.4%	2.7%
≥ 10	0.2%	0.01%	0.02%	0.3%	0.02%	0.03%
Age						
Mean age (in years)	56.0 (SD 17.6)	46.8 (SD 16.8)	47.1 (SD 16.9)	53.2 (SD 16.7)	45.8 (SD 16.3)	45.9 (SD 16.3)
Categories	
18–44 years	*N* = 7340 2.3%	*N* = 312,600 97.7%	*N* = 319,940 100%	*N* = 4411 1.4%	*N* = 303,200 98.6%	*N* = 307,611 100%
45–64 years	*N* = 8850 3.8%	*N* = 221,751 96.2%	*N* = 230,601 100%	*N* = 5393 2.4%	*N* = 216,282 97.6%	*N* = 221,675 100%
65–79 years	*N* = 7510 7.0%	*N* = 100,191 93.0%	*N* = 107,701 100%	*N* = 3372 3.7%	*N* = 86,854 96.3%	*N* = 90,226 100%
80+ years	*N* = 2185 11.3%	*N* = 17,225 88.7%	*N* = 19,410 100%	*N* = 672 6.4%	*N* = 9859 93.6%	*N* = 10,531 100%
Socioeconomic status Proportion of participants in mosaic Groups 1, 2, and 3^a^ (in percent)	1: 28.6%	1: 30.6%	1: 30.6%	1: 30.2%	1: 30.5%	1: 30.5%
	2: 36.4%	2: 39.3%	2: 38.9%	2: 36.1%	2: 39.8%	2: 39.7%
	3: 34.3%	3: 29.2%	3: 29.4%	3: 32.6%	3: 28.8%	3: 28.9%
Number of visits in primary care^b^ (mean number (*N*) and (standard deviation (SD)))	4.3 (SD 5.1)	2.2 (SD 3.2)	2.3 (SD 3.3)	3.5 (SD 4.8)	1.5 (SD 3.0)	1.6 (SD 3.0)
Number of visits in secondary care^b^ (mean number (*N*) and (standard deviation (SD)))	5.3 (SD 6.9)	2.2 (SD 3.9)	2.3 (SD 4.1)	4.6 (SD 7.7)	1.5 (SD 3.8)	1.6 (SD 4.0)
Number of hospital admissions (mean number (*N*) and (standard deviation (SD)))	0.5 (SD 1.4)	0.1 (SD 0.6)	0.2 (SD 0.6)	0.5 (SD 1.6)	0.1 (SD 0.6)	0.1 (SD 0.6)
Proportion of participants with ≥ 1 psychiatric diagnoses^c^ (in percent)	69.4%	26.5%	28.1%	65.0%	17.0%	18.1%
Proportion of participants with polypharmacy^d^ (in percent)	84.8%	36.8%	38.6%	76.0%	25.1%	26.2%
Proportion of collected prescriptions of benzodiazepines prescribed in (in percent):						
Primary care (P)	P: 57.3%			P: 55.9%		
Secondary care (S)	S: 26.3%			S: 28.2%		
Both (B)	B: 16.4%			B: 16.0%		

*Note:* Descriptive data analysis of all individuals with and without ≥ 2 collected prescriptions of benzodiazepines from January 1, 2018, until December 31, 2019.

a Mosaic Group 1 is defined as having high socioeconomic status, Group 2 to have intermediate socioeconomic status, and Group 3 to have low socioeconomic status [35, 36].

b Defined as visits to a physician.

c Phsyciatric diagnoses defined according to ICD‐10, Chapter F.

d Participants with ≥ 5 collected prescriptions of medications.

Of the total population of 1,343,637 individuals during COVID‐19 pandemic, 21,280 women (3.1%) and 10,963 men (1.8%) had ≥ 2 collected benzodiazepine prescriptions. Similar findings as described for the population before COVID‐19 pandemic were seen in the population with ≥ 2 collected benzodiazepine prescriptions during COVID‐19 pandemic (Table 2). In the washout analysis, the number of individuals with ≥ 2 collected benzodiazepine prescriptions decreased slightly (Supporting Information S3: Table 2a,b).

**Table 2:** Baseline data of individuals with and without ≥ 2 collected prescriptions of benzodiazepines during the COVID‐19 pandemic.

	Women			Men		
	≥ 2 collected prescriptions of Benzodiazepines			≥ 2 collected prescriptions of Benzodiazepines		
	Yes	No	Total	Yes	No	Total
Total number (*N*) and percent of patients with or without ≥ 2 collected prescriptions of benzodiazepines	*N* = 21,280 3.1%	*N* = 673,904 96.9%	*N* = 695,184 100%	*N* = 10,963 1.8%	*N* = 637,156 98.2%	*N* = 648,119 100%
Number of chronic diagnoses	
Mean number	2.64 (SD 1.90)	1.03 (SD 1.34)	1.08 (SD 1.39)	2.48 (SD 2.04)	0.93 (SD 1.39)	0.95 (SD 1.42)
Categories	
0–1	30.8%	73.0%	71.7%	37.5%	76.2%	75.5%
2	22.1%	13.9%	14.2%	20.0%	11.3%	11.5%
3	18.7%	7.1%	7.4%	15.7%	6.2%	6.3%
4	12.5%	3.3%	3.6%	11.6%	3.3%	3.4%
5–9	15.6%	2.6%	3.0%	14.7%	3.0%	3.2%
≥ 10	0.3%	0.02%	0.03%	0.5%	0.03%	0.04%
Age						
Mean age (in years)	56.8 (SD 17.4)	47.0 (SD 16.8)	47.3 (SD 16.9)	53.7 (SD 16.7)	46.1 (SD 16.3)	46.2 (SD 16.3)
Categories	
18–44 years	*N* = 5767 1.8%	*N* = 319,798 98.2%	*N* = 325,565 100%	*N* = 3549 1.4%	*N* = 310,190 98.6%	*N* = 313,739 100%
45–64 years	*N* = 7393 3.1%	*N* = 229,304 96.9%	*N* = 236,697 100%	*N* = 4395 1.9%	*N* = 223,922 98.1%	*N* = 228,317 100%
65–79 years	*N* = 6689 5.9%	*N* = 106,781 94.1%	*N* = 113,470 100%	*N* = 2871 3.0%	*N* = 92,251 97.0%	*N* = 95,122 100%
80+ years	*N* = 1845 9.5%	*N* = 17,607 90.5%	*N* = 19,452 100%	*N* = 561 5.1%	*N* = 10,380 94.9%	*N* = 10,941 100%
Socioeconomic status Proportion of participants in mosaic Groups 1, 2, and 3^a^ (in percent)	1: 28.3%	1: 30.6%	1: 30.5%	1: 29.9%	1: 30.4%	1: 30.4%
	2: 37.0%	2: 39.7%	2: 39.6%	2: 37.2%	2: 40.1%	2: 40.1%
	3: 34.4%	3: 29.4%	3: 30.0%	3: 32.1%	3: 29.1%	3:29.1%
Number of visits in primary care^b^ (mean number (*N*) and (standard deviation (SD)))	4.1 (SD 4.6)	2.0 (SD 2.9)	2.1 (SD 3.0)	3.3 (SD 4.2)	1.4 (SD 2.6)	1.5 (SD 2.6)
Number of visits in secondary care^b^ (mean number (*N*) and (standard deviation (SD)))	5.3 (SD:7)	2.2 (SD 3.8)	2.3 (SD 4.0)	4.5 (SD 8.1)	1.5 (SD 3.9)	1.6 (SD 4.0)
Number of hospital admissions (mean number (*N*) and (standard deviation (SD)))	0.5 (SD 1.4)	0.1 (SD 0.5)	0.1 (SD 0.6)	0.5 (SD 1.5)	0.1 (SD 0.6)	0.1 (SD 0.6)
Proportion of participants with ≥ 1 psychiatric diagnoses^c^ (in percent)	71.9%	28.5%	29.8%	67.5%	18.5%	19.3%
Proportion of participants with polypharmacy^d^ (in percent)	86.9%	37.4%	38.9%	78.3%	25.7%	26.7%
Proportion of collected prescriptions of benzodiazepines prescribed in (in percent):						
Primary care (P)	P: 58.3%			P: 56.0%		
Secondary care (S)	S: 27.0%			S: 29.8%		
Both (B)	B: 14.7%			B: 14.2%		

*Note:* Descriptive data analysis of all individuals with and without ≥ 2 collected prescriptions of benzodiazepines from January 1, 2020, until December 31, 2021.

a Mosaic Group 1 is defined as having high socioeconomic status, Group 2 to have intermediate socioeconomic status, and Group 3 to have low socioeconomic status [35, 36].

b Defined as visits to a physician.

c Phsyciatric diagnoses defined according to ICD‐10, Chapter F.

d Participants with ≥ 5 collected prescriptions of medications.

### 3.2 Logistic Regression Models

The association of having ≥ 2 collected benzodiazepine prescriptions significantly increased for individuals with two chronic conditions compared to the reference group for both men and women in both cohorts. OR 3.57 (CI 3.57–3.58) for men and 3.25 (CI 3.25–3.25) for women before the COVID‐19 pandemic, and OR 3.36 (CI 3.36–3.37) for men and 3.41 (CI 3.24–3.59) for women during the COVID‐19 pandemic. In addition, the association increased with an increased number of chronic conditions for both men and women and in both cohorts. OR 13.26 (CI 13.25–13.28) for men, and 11.41 (CI 11.40–11.42) for women with 5–9 chronic conditions before the COVID‐19 pandemic, and OR 12.28 (CI 12.26–12.30) for men and 11.18 (CI 10.48–11.93) for women with 5–9 chronic conditions during the COVID‐19 pandemic (Table 3 and Supporting Information S1: Figure 2).

**Table 3:** Potentially inappropriate benzodiazepine prescribing before and during COVID‐19 pandemic.

Number of chronic conditions	Women, OR (99% CI)		Men, OR (99% CI)	
	Before COVID‐19	During COVID‐19	Before COVID‐19	During COVID‐19
2	3.25 (3.25–3.25)	3.41 (3.24–3.59)	3.57 (3.57–3.58)	3.36 (3.36–3.37)
3	5.30 (5.29–5.30)	5.41 (5.11–5.72)	5.11 (5.10–5.12)	4.93 (4.92–4.94)
4	9.02 (9.01–9.03)	7.38 (6.91–7.89)	7.42 (7.41–7.44)	7.27 (7.26–7.29)
5–9	11.41 (11.40–11.42)	11.18 (10.48–11.93)	13.26 (13.25–13.28)	12.28 (12.26–12.30)
≥ 10	NA^a^	24.37 (16.43–36.14)	NA^a^	NA^a^

*Note:* Logistic regression model. The outcome measurement *potentially inappropriate prescribing* was defined as ≥ 2 collected prescriptions of benzodiazepines during 2 years of time before, January 1, 2018, until December 31, 2019, and during COVID‐19 pandemic, January 1, 2020, until December 31, 2021. Exposure was number of chronic conditions the 4 previous years to outcome measurement, January 1, 2014, until December 31, 2017, and January 1, 2016, until December 31, 2019, respectively. Reference was 0–1 chronic disease. The logistic regression model was adjusted for age as a continuous variable in years, and socioeconomic status as a categoric variable using Mosaic areas, representing neighborhoods with high (1), medium (2), and low (3) socioeconomic status [35, 36].

a Not available.

The associations for men and women before and during COVID‐19 pandemic decreased but remained significant when adjusted for each of the clinical confounders in additional models. In addition, the associations remained at similar levels in the washout analysis for both men and women before and during COVID‐19 pandemic (Supporting Information S4: Table 3).

Due to missing values for addresses, 0.8% of all individuals before and 0.3% of all individuals during COVID‐19 pandemic were deleted from the data analysis.

## 4 Discussion

In this total population cohort study, we identified an association between having ≥ 2 collected prescriptions of benzodiazepines and an increasing number of chronic conditions in both men and women in Region Stockholm, Sweden. This association was seen in two cohorts, both before and during the COVID‐19 pandemic. All associations remained significant but were attenuated in both cohorts when adjusted for the potential confounders: number of visits in primary and secondary care, having a psychiatric diagnosis, and polypharmacy. In addition, the association regarded both continuous and new prescribing of benzodiazepines.

### 4.1 Strengths and Limitations

A strength of this study was the use of a total population database with very little loss to follow‐up, decreasing the risk of selection bias. A further strength of the study design was that the logistic regression model was adjusted for clinically important confounders [19, 39]. In addition, in our subgroup analysis we identified similar associations in individuals who had not been prescribed benzodiazepines in the 2 years previous to the outcome measurement, compared to all individuals (Supporting Information S3 and S4: Tables 2a,b and 3).

An additional strength was the large population. In the logistic regression analysis of OR with 99% confidence intervals, several confidence intervals appeared narrow. Because the data were reported to two decimal places, one confidence interval was displayed as having zero width. If three decimal places had been used, the interval would still have been narrow but visibly wider. These findings are interpreted as being related to the large number of exposed individuals with the outcome. The number of participants with the outcome in this study is substantially higher than in many comparable studies.

A limitation of this study was that the study design could only follow‐up groups of individuals rather than separate individuals. Hence, we were unable to address causality and draw statistical conclusions of incidence. Instead, statistical inferences were made based on associations. However, to improve the internal validity of the study design, the previously mentioned subgroup analysis was conducted.

This study has limitations related to information bias. The primary outcome was intended to capture potentially inappropriate prescribing, but observational data cannot confirm appropriateness. Our measure did not account for dose or indication, so some appropriate use may have been included. Indication data were unavailable; excluding patients with epilepsy or dementia could have reduced misclassification but would also remove relevant cases. Nursing home status and behavioral symptoms of dementia were similarly difficult to assess. We did not use DDD, as benzodiazepines are not recommended for continuous use; if lower doses were preferred, a DDD cutoff could have been appropriate. Another limitation is the lack of consensus on counting chronic conditions in multimorbidity research, affecting generalizability. We applied an international list of 40 conditions adapted for Sweden (Supporting Information S2: Table 1). During analysis, two new definitions were published, both supporting the concept that condition count reflects the severity of multimorbidity, which aligns with our approach [7, 8].

Another risk for information bias in this study is the risk of exposure misclassification. Individuals with a higher number of chronic conditions have more healthcare contacts and therefore risk having more medications prescribed, which could lead to overestimation of the outcome measurement. However, the original logistic regression model was adjusted for number of healthcare contacts to address this risk. The associations were attenuated but remained significant.

This study has potential confounder bias. Due to data limitations, the logistic regression model could not be adjusted for the known social determinants for benzodiazepine prescribing living alone, having low education level, or low income [19]. The only available socioeconomic measure was Mosaic, a measure validated to reflect socioeconomic status of the area in which an individual resides but that does not give granular data about an individual's actual socioeconomic status, income, or level of education [35, 36]. Mosaic areas may include individuals with low socioeconomic status living in high‐status areas and vice versa. This potential bias likely affects both groups equally, regardless of the outcome measure. Moreover, people with lower socioeconomic status in higher‐status areas may adopt healthier habits, and the reverse may also occur, reducing the risk of bias. We had no data on household size. Polypharmacy was defined as ≥ 5 collected prescriptions. However, assessing prescriptions at a single point in time may overestimate polypharmacy. This possible overestimation would apply to both patients with and without the outcome, minimizing the risk of assessment bias.

### 4.2 Comparison With Previous Studies

The results are in agreement with previous studies showing an association between MLTC and benzodiazepine prescribing in similar settings [18, 19]. In addition, the results are in agreement with the findings of an association between increased number of chronic conditions and increased prescriptions of both benzodiazepines and Z‐drugs for older adults in French primary care [26]. The current study adds evidence for a dose–response pattern of association between an increasing number of chronic conditions and risk of, in particular, benzodiazepine prescriptions in both primary and secondary care in a total adult population. No previous study to our knowledge has explored this association before. The findings also indirectly support previous discussions of the number of chronic conditions important for evaluating MLTC complexity [7, 8].

Psychiatric disease, polypharmacy, and increasing number of visits in primary and secondary care were all associated with an increased prevalence of potentially inappropriate benzodiazepine prescribing in this study, as previously reported [19, 39]. These clinical factors attenuated but did not explain all the significant results of this study. We found only a tendency toward significant association between lower socioeconomic status and benzodiazepine prescribing (Tables 1 and 2), unlike results from other contexts [19]. This might be due to different definitions of socioeconomic status used in different studies, or because the association might be less strong in a Swedish context.

We describe in this study that prescribing rate of benzodiazepines remained at the same levels or with slightly lower levels during the COVID‐19 pandemic than before. However, comparing the two cohorts was not an aim of this study and no statistical inferences can be made from this description. In other parts of the world, benzodiazepine prescribing increased during the pandemic, perhaps reflecting different healthcare strategies internationally [40, 41].

### 4.3 Implications

In this study, we found that individuals with a higher number of chronic conditions face an increased risk of receiving two or more benzodiazepine prescriptions, both as ongoing and new prescriptions. These findings should be interpreted with caution, as we were unable to assess the appropriateness of prescribing in this study. Further research is needed to better understand this relationship. Nevertheless, the results can inform policymakers and healthcare providers about the heightened risk of benzodiazepine prescribing among populations with MLTC. This underscores the need to develop strategies aimed at reducing prescribing in these individuals. Potential strategies may include implementing multidisciplinary care models, enhancing prescriber education, and promoting deprescribing initiatives.

## 5 Conclusions

In this study, individuals with an increasing number of chronic conditions were more likely to receive both ongoing and newly initiated benzodiazepine prescriptions. While the appropriateness of prescribing was not assessed in this study, these findings can inform healthcare providers and policymakers about the need for strategies to reduce such prescribing in this population.

## Author Contributions

**Caroline Kappelin:** conceptualization, formal analysis, project administration, writing – original draft, writing – review and editing, methodology. **Caroline Wachtler:** conceptualization, methodology, supervision, writing – review and editing. **Gunnar Ljunggren:** conceptualization, methodology, software, data curation, formal analysis, writing – review and editing. **Axel C. Carlsson:** conceptualization, methodology, software, data curation, formal analysis, supervision, writing – review and editing. All authors have read and approved the final version of the manuscript.

## Ethics Statement

This study was performed in line with the principles of the Declaration of Helsinki. The study was approved by the Swedish Ethics Committee (Registration Number 2021‐01016).

## Consent

Individual consent was not applicable due to registry‐based data.

## Conflicts of Interest

The authors declare no conflicts of interest.

## 1 Transparency Statement

First author Caroline Kappelin affirms that this manuscript is an honest, accurate, and transparent account of the study being reported; that no important aspects of the study have been omitted; and that any discrepancies from the study as planned have been explained.

## Supporting information


Supporting File 1

Supporting File 2

Supporting File 3

Supporting File 4

## Data Availability

The data used in this study can be made available for research upon ethical approval and request via halsodata.rst@regionstockholm.se. The first author had full access to all the data in this study and takes complete responsibility for the integrity of the data and the accuracy of the data analysis.

## References

[1] M. Fortin , M. Stewart , M. E. Poitras , J. Almirall , and H. Maddocks , “A Systematic Review of Prevalence Studies on Multimorbidity: Toward a More Uniform Methodology,” Annals of Family Medicine 10, no. 2 (March 2012): 142–151. 22412006 10.1370/afm.1337 PMC3315131

[2] T. T. Makovski , S. Schmitz , M. P. Zeegers , S. Stranges , and M. van den Akker , “Multimorbidity and Quality of Life: Systematic Literature Review and Meta‐Analysis,” Ageing Research Reviews 53 (August 2019): 100903. 31048032 10.1016/j.arr.2019.04.005

[3] J. R. Read , L. Sharpe , M. Modini , and B. F. Dear , “Multimorbidity and Depression: A Systematic Review and Meta‐Analysis,” Journal of Affective Disorders 221 (October 2017): 36–46. 28628766 10.1016/j.jad.2017.06.009

[4] R. El‐Gabalawy , C. S. Mackenzie , S. Shooshtari , and J. Sareen , “Comorbid Physical Health Conditions and Anxiety Disorders: A Population‐Based Exploration of Prevalence and Health Outcomes Among Older Adults,” General Hospital Psychiatry 33, no. 6 (December 2011): 556–564. 21908055 10.1016/j.genhosppsych.2011.07.005

[5] K. Kroenke , J. L. Jackson , and J. Chamberlin , “Depressive and Anxiety Disorders in Patients Presenting With Physical Complaints,” American Journal of Medicine 103, no. 5 (November 1997): 339–347. 9375700 10.1016/s0002-9343(97)00241-6

[6] J. E. Wilk , J. C. West , W. E. Narrow , et al., “Comorbidity Patterns in Routine Psychiatric Practice: Is There Evidence of Underdetection and Underdiagnosis?,” Comprehensive Psychiatry 47, no. 4 (July 2006): 258–264. 16769299 10.1016/j.comppsych.2005.08.007

[7] I. S. S. Ho , A. Azcoaga‐Lorenzo , A. Akbari , et al., “Measuring Multimorbidity in Research: Delphi Consensus Study,” BMJ Medicine 1, no. 1 (July 2022): e000247, https://bmjmedicine.bmj.com/content/1/1/e000247. 36936594 10.1136/bmjmed-2022-000247 PMC9978673

[8] V. Kuan , S. Denaxas, P. Patalay, et al., “Identifying and Visualising Multimorbidity and Comorbidity Patterns in Patients in the English National Health Service: A Population‐Based Study,” *Lancet Digital Health* 5 (2022): e16‐e27. 10.1016/S2589-7500(22)00187-X 36460578

[9] C. May , V. M. Montori , and F. S. Mair , “We Need Minimally Disruptive Medicine,” BMJ 339 (August 2009): b2803. 19671932 10.1136/bmj.b2803

[10] World Health Organization , Medication Safety in Polypharmacy: Technical Report (World Health Organization, 2019), https://apps.who.int/iris/handle/10665/325454.

[11] T. Forslund , A. C. Carlsson , G. Ljunggren , J. Ärnlöv , and C. Wachtler , “Patterns of Multimorbidity and Pharmacotherapy: A Total Population Cross‐Sectional Study,” Family Practice 38, no. 2 (2021): 132–140. 10.1093/fampra/cmaa056. 32766818 PMC8006765

[12] A. Nobili , A. Marengoni , M. Tettamanti , et al., “Association Between Clusters of Diseases and Polypharmacy in Hospitalized Elderly Patients: Results From the REPOSI Study,” European Journal of Internal Medicine 22, no. 6 (December 2011): 597–602. 22075287 10.1016/j.ejim.2011.08.029

[13] M. S. Dimitrow , M. S. A. Airaksinen , S. L. Kivelä , A. Lyles , and S. N. S. Leikola , “Comparison of Prescribing Criteria to Evaluate the Appropriateness of Drug Treatment in Individuals Aged 65 and Older: A Systematic Review,” Journal of the American Geriatrics Society 59, no. 8 (2011): 1521–1530. 21797829 10.1111/j.1532-5415.2011.03497.x

[14] M. H. Beers , “Explicit Criteria for Determining Potentially Inappropriate Medication Use by the Elderly: An Update,” Archives of Internal Medicine 157, no. 14 (July 1997): 1531–1536. 9236554

[15] D. O'Mahony , D. O'Sullivan , S. Byrne , M. N. O'Connor , C. Ryan , and P. Gallagher , “STOPP/START Criteria for Potentially Inappropriate Prescribing in Older People: Version 2,” Age and Ageing 44, no. 2 (March 2015): 213–218. 25324330 10.1093/ageing/afu145 PMC4339726

[16] H. M. Holmes , R. Luo , Y. F. Kuo , J. Baillargeon , and J. S. Goodwin , “Association of Potentially Inappropriate Medication Use With Patient and Prescriber Characteristics in Medicare Part D,” Pharmacoepidemiology and Drug Safety 22, no. 7 (July 2013): 728–734. 23494811 10.1002/pds.3431 PMC3701724

[17] A. Spinewine , K. E. Schmader , N. Barber , et al., “Appropriate Prescribing in Elderly People: How Well Can It Be Measured and Optimised?,” Lancet 370, no. 9582 (July 2007): 173–184. 17630041 10.1016/S0140-6736(07)61091-5

[18] K. Linnet , L. S. Gudmundsson , F. G. Birgisdottir , et al., “Multimorbidity and Use of Hypnotic and Anxiolytic Drugs: Cross‐Sectional and Follow‐Up Study in Primary Healthcare in Iceland,” BMC Family Practice 17 (June 2016): 69, https://www.ncbi.nlm.nih.gov/pmc/articles/PMC4896036/. 27267943 10.1186/s12875-016-0469-0 PMC4896036

[19] L. Manthey , T. van Veen , E. J. Giltay , et al., “Correlates of (Inappropriate) Benzodiazepine Use: The Netherlands Study of Depression and Anxiety (NESDA),” British Journal of Clinical Pharmacology 71, no. 2 (February 2011): 263–272. 21219408 10.1111/j.1365-2125.2010.03818.x PMC3040548

[20] Swedish Medical Products Agency, *Recommendations for Treatment of Depression, Anxiety in Children and Adults* (Swedish Medical Products Agency, 2016), https://www.lakemedelsverket.se/sv/behandling-och-forskrivning/behandlingsrekommendationer/sok-behandlingsrekommendationer/lakemedel-vid-depression-angestsyndrom-och-tvangssyndrom-hos-barn-och-vuxna---behandlingsrekommendation.

[21] M. Lader , “Benzodiazepines Revisited—Will We Ever Learn?,” Addiction 106, no. 12 (2011): 2086–2109. 21714826 10.1111/j.1360-0443.2011.03563.x

[22] R. M. C. Herings , B. H. Stricker , A. de Boer , A. Bakker , and F. Sturmans , “Benzodiazepines and the Risk of Falling Leading to Femur Fractures: Dosage More Important Than Elimination Half‐Life,” Archives of Internal Medicine 155, no. 16 (1995): 1801–1807. 7654115

[23] M. J. Barker , K. M. Greenwood , M. Jackson , and S. F. Crowe , “Cognitive Effects of Long‐Term Benzodiazepine Use: A Meta‐Analysis,” CNS Drugs 18, no. 1 (2004): 37–48. 14731058 10.2165/00023210-200418010-00004

[24] G. M. Brophy , R. Bell , J. Claassen , et al. “Guidelines for the Evaluation and Management of Status Epilepticus,” Neurocrit Care 17 (2012): 3–23. 22528274 10.1007/s12028-012-9695-z

[25] O. P. Tible , F. Riese , E. Savaskan , and A. von Gunten , “Best Practice in Management of Behavioural and Psychological Symptoms of Dementia,” Therapeutic Advances in Neurological Disorders 10, no. 8 (2017): 297–309. 28781611 10.1177/1756285617712979 PMC5518961

[26] J. Yana , L. Moscova , J. Le Breton , et al., “Prescription of Benzodiazepines and Z‐Drugs Among Older Patients in Primary Care: A French, National, Cohort Study,” Family Practice 41, no. 4 (2024): 419–425. 36308516 10.1093/fampra/cmac114

[27] World Health Organisation , Innovative Care for Chronic Conditions: Building Blocks for Action: Global Report (WHO, 2002).

[28] STROBE, “STROBE Checklist,” cited November 10, 2023, https://www.strobe-statement.org/checklists/.

[29] L. D. Delaney , M. C. Bicket , H. M. Hu , et al., “Opioid and Benzodiazepine Prescribing After COVID‐19 Hospitalization,” Journal of Hospital Medicine 17, no. 7 (July 2022): 539–544. 35621024 10.1002/jhm.12842 PMC9347718

[30] A. C. Carlsson , P. Wändell , U. Ösby , R. Zarrinkoub , B. Wettermark , and G. Ljunggren , “High Prevalence of Diagnosis of Diabetes, Depression, Anxiety, Hypertension, Asthma and COPD in the Total Population of Stockholm, Sweden – A Challenge for Public Health,” BMC Public Health 13, no. 1 (July 2013): 670. 23866784 10.1186/1471-2458-13-670 PMC3724714

[31] K. Barnett , S. W. Mercer , M. Norbury , G. Watt , S. Wyke , and B. Guthrie , “Epidemiology of Multimorbidity and Implications for Health Care, Research, and Medical Education: A Cross‐Sectional Study,” Lancet 380, no. 9836 (July 2012): 37–43. 22579043 10.1016/S0140-6736(12)60240-2

[32] C. Harrison , H. Britt , G. Miller , and J. Henderson , “Examining Different Measures of Multimorbidity, Using a Large Prospective Cross‐Sectional Study in Australian General Practice,” BMJ Open 4, no. 7 (July 2014): e004694. 10.1136/bmjopen-2013-004694 PMC4120329 25015470

[33] A. Head , K. Fleming , C. Kypridemos , P. Schofield , J. Pearson‐Stuttard , and M. O'Flaherty , “Inequalities in Incident and Prevalent Multimorbidity in England, 2004–19: A Population‐Based, Descriptive Study,” Lancet Healthy Longevity 2, no. 8 (August 2021): e489–e497. 36097998 10.1016/S2666-7568(21)00146-X

[34] A. Kingston , L. Robinson , H. Booth , M. Knapp , and C. Jagger , “Projections of Multi‐Morbidity in the Older Population in England to 2035: Estimates From the Population Ageing and Care Simulation (PACSim) Model,” Age and Ageing 47, no. 3 (May 2018): 374–380. 29370339 10.1093/ageing/afx201 PMC5920286

[35] A. Sharma , S. Lewis , and L. Szatkowski , “Insights Into Social Disparities in Smoking Prevalence Using Mosaic, a Novel Measure of Socioeconomic Status: An Analysis Using a Large Primary Care Dataset,” BMC Public Health 10, no. 1 (December 2010): 755. 21138555 10.1186/1471-2458-10-755 PMC3016386

[36] L. Douglas and L. Szatkowski , “Socioeconomic Variations in Access to Smoking Cessation Interventions in UK Primary Care: Insights Using the Mosaic Classification in a Large Dataset of Primary Care Records,” BMC Public Health 13 (June 2013): 546. 23738743 10.1186/1471-2458-13-546 PMC3710237

[37] D. G. Kleinbaum and M. Klein , Logistic Regression. Statistics for Biology and Health (Springer, 2010), http://link.springer.com/10.1007/978-1-4419-1742-3.

[38] “Substance Abuse Among Older Adults – ClinicalKey,” cited December 14, 2022, https://www-clinicalkey-com.sll.idm.oclc.org/#!/content/playContent/1-s2.0-S0749069014000433?returnurl=https:%2F%2Flinkinghub.elsevier.com%2Fretrieve%2Fpii%2FS0749069014000433%3Fshowall%3Dtrue&referrer=https:%2F%2Flink.springer.com%2F.

[39] A. Kuerbis , P. Sacco , D. G. Blazer , and A. A. Moore , “Substance Abuse Among Older Adults,” Clinics in Geriatric Medicine 30, no. 3 (August 2014): 629–654. 25037298 10.1016/j.cger.2014.04.008 PMC4146436

[40] I. Bužančić , T. I. Pejaković , and M. O. Hadžiabdić , “A Need for Benzodiazepine Deprescribing in the COVID‐19 Pandemic: A Cohort Study,” Pharmacy 10, no. 5 (September 2022): 120. 36287441 10.3390/pharmacy10050120 PMC9611451

[41] A. Sarangi , T. McMahon , and J. Gude , “Benzodiazepine Misuse: An Epidemic Within a Pandemic,” Cureus 13, no. 6 (2021): e15816. 34306882 10.7759/cureus.15816 PMC8294026

