# Supplementary material for: The Association Between Number of Chronic Conditions and Benzodiazepine Prescribing in Region Stockholm, Sweden: A Total Population‐Based Cohort Study
Source: Health Sci Rep. 2026 Aug 9;9(8):e72970. doi: 10.1002/hsr2.72970 (PMC13454220; doi:10.1002/hsr2.72970)
Supplement: Supplementary file 1 [file HSR2-9-e72970-s001.docx]

Supplementary Material

**Figure 2.** Odds ratios of ≥2 collected prescriptions of benzodiazepines with an increasing number of chronic conditions for women and men before, January 1, 2018, until December 31, 2019, and during COVID-19, January 1, 2020, until December 31, 2021
