# Supplementary material for: The Association Between Number of Chronic Conditions and Benzodiazepine Prescribing in Region Stockholm, Sweden: A Total Population‐Based Cohort Study
Source: Health Sci Rep. 2026 Aug 9;9(8):e72970. doi: 10.1002/hsr2.72970 (PMC13454220; doi:10.1002/hsr2.72970)
Supplement: Supplementary file 2 [file HSR2-9-e72970-s002.docx]

Supplementary Material

# Table 1. Definitions of 40 chronic health conditions

| **Name** | **Definition** | **ICD** | **Condition** |
| --- | --- | --- | --- |
| **Alcohol problems** | ICD code ever | F10 | Alcohol dependency |
| **Asthma (current)** | ICD code in past year | J45  J46J45 | Asthma  Acute asthma |
| **Atrial fibrillation** | ICD code ever | I48 | Atrial fibrillation |
| **Anorexia or bulimia** | ICD code ever | F50 | Eating disorders |
| **Anxiety & other neurotic, stress related & somatoform disorders** | ICD code in past year | F40  F41  F42  F43 | Phobias  Anxiety disorders  OCD  Stress-related disorders |
| **Blindness & low vision** | ICD code ever | H54 | Loss of sight |
| **Bronchiectasis** | ICD code ever | J47 | Bronchiectasis |
| **Cancer (new diagnosis in last five years)** | ICD code in past 5 years | C01-C97 | Malign tumors |
| **Chronic liver disease** | ICD code ever | K70  K72.1  K72.9  K74  K75  K76  K77 | Alcohol-related liver disease  Chronic liver failure  Unspecified liver failure  Cirrhosis  Other specified liver disease  Unspecified liver disease  Secondary liver disease |
| **Chronic obstructive pulmonary disease** | ICD code ever | J41  J42  J43  J44 | Specified chronic bronchitis  Unspecified chronic bronchitis  Emphysema  COPD |
| **Chronic renal disease** | ICD code ever | N18  N19  I12 | Chronic renal disease  Unspecified chronic renal disease  Hypertensive renal disease w/ renal failure |
| **Chronic sinusitis** | ICD code ever | J32 | Chronic sinusitis |
| **Constipation (current)** | ICD code in past year | K59.0 | Constipation |
| **Coronary heart disease** | ICD code ever | I20  I21  I22  I23  I24  I25 | Angina pectoris  Myocardial infarction  Reinfarction  Complications to MI  Acute ischemic heart disease  Chronic ischemic heart disease |
| **Dementia** | ICD code ever | F00  F01  F02  F03 | Alzheimer’s dementia  Vascular dementia  Secondary dementia  Unspecified dementia |
| **Depression** | ICD code in past year | F32  F33  F34  F38  F39 | Depressive episodes  Recurrent depression  Persistent mood disorders  Other mood disorders  Unspecified mood disorders |
| **Diabetes** | ICD code ever | E10  E11  E12  E13  E14 | DM type 1  DM type 2  Starvation related DM  Other specified DM  Unspecified DM |
| **Diverticular disease of intestine** | ICD code ever | K52 | Diverticular disease |
| **Dyspepsia (current)** | ICD code in past year | K30  K29.7  K29.6  K29.5  K29.4  K 29.3 | Dyspepsia  Unspecified gastritis  Other specified gastritis  Chronic unspecified gastritis  Chronic atrophic gastritis  Chronic superficial gastritis |
| **Epilepsy (current)** | ICD code in past year | G40 | Epilepsy |
| **Glaucoma** | ICD code ever | H40  H42 | Primary glaucoma  Secondary glaucoma |
| **Hearing loss** | ICD code ever | H91  H92 | Specified hearing loss.  General hearing loss |
| **Heart failure** | ICD code ever | I110  I50 | Hypertensive heart disease w/ heart failure  Heart failure |
| **Hypertension** | ICD code ever | I10  I119  I12  I13  I15 | Essential hypertension,  Hypertensive heart disease,  Hypertensive kidney disease,  Hypertensive heart and kidney disease  Secondary hypertension |
| **Inflammatory bowel disease** | ICD code ever | K50  K51 | Crohn’s disease  Ulcerous colitis |
| **Irritable bowel syndrome** | ICD code ever | K58 | Colon irritable |
| **Learning disability** | ICD code ever | F81 | Learning disabilities |
| **Migraine (current)** | ICD code in past year | G43 | Migraine |
| **Multiple sclerosis** | ICD code ever | G359 | Multiple sclerosis |
| **Painful condition** | ICD code in past year | K580  K589  M797  R521  R522  R522A  R522B  R522C B022 G500  E104 E114 E124 E134 E144  G099 G359  G500 G508 G509 G530 G540 G541 G542 G543 G544 G545 G546 G548 G549  G551 G552 G553 G558 G560 G561 G562 G562B G562C G562D G562X G563 G563B G563C G563D G563X  G564 G568 G569 G570 G571 G572 G573 G574 G575 G576 G578 G579 G580 G587 G588 G589 G590 G598  G600 G601 G602 G603 G608 G609 G618 G619 G620 G621 G622 G628 G629 G630 G631 G632 G633 G634  G635 G636 G638  G952  B02- B91- G35- G500 G546 G549P G560 G573 G58-P G621 G629P I69-  E108P E118P F454  B02- B91- G35- G500 G546 G549P G560 G573 G58-P G621 G629P I69- | Irritable bowel with diarrhea  Irritable bowel without diarrhea  Fibromyalgia  Chronic treatment resistant pain  Other chronic pain  Persistent pain, nociceptive  Persistent pain, neuropathic  Persistent pain, of unclear etiology |
| **Parkinson’s disease** | ICD code ever | G20 | Parkinson’s disease |
| **Peripheral vascular disease** | ICD code ever | I73 | All peripheral vascular disease (including I73.0) |
| **Prostate disorders** | ICD code ever | N40  N41  N42 | Prostate enlargement  Prostatitis  Other prostate disease |
| **Psoriasis or eczema** | ICD code in past year | L20  L23  L24  L25  L26  L30  L40 | Allergic eczema  Allergic contact eczema  Irritative contact eczema  Unspecified contact eczema  Exfoliative dermatitis  Eczema  Psoriasis |
| **Rheumatoid arthritis, other inflammatory polyarthropathies & systematic connective tissue disorders** | ICD code ever | M05  M06  M07  M08  M09  M10  M11  M12  M30  M31  M32  M33  M34  M35 | Seropositive rheumatoid arthritis  Rheumatoid arthritis  Psoriasis arthropathy  Juvenile rheumatoid arthritis  Juvenile arthritis + other disease  Gout  Pyrophosphate arthritis  Other synovitis  Specific polyarthritis  Specific systemic tissue disorders  SLE  Myositis  Sclerosis  Other systemic tissue disorders |
| **Schizophrenia (and related non-organic psychosis) or bipolar disorder** | ICD code in past year | F20  F30  F31 | Schizophrenia  Bipolar disorder (mania)  Bipolar disorder (depression) |
| **Stroke & transient ischemic attack** | ICD code ever | I60  I61  I62  I63  I64  I65    I66    I67  I68  I69 | Subarachnoid hemorrhage  Intracerebral hemorrhage  Subdural hemorrhage  Cerebral infarction  Acute cerebrovascular disease  Occlusion and stenosis of precerebral arteries  Occlusion and stenosis of intracranial arteries  Aneurysm  Other cerebrovascular disease  Late effects of cerebrovascular disease |
| **Substance misuse (psychoactive not alcohol)** | ICD code ever | F11  F12  F13  F14  F15  F16  F18  F19 | Opioids  Cannabis  Sedatives/ hypnotics  Cocaine  Stimulants  Hallucinogens  Solvents  Combinations |
| **Thyroid disorders** | ICD code ever | E01  E02  E03  E04  E05  E06  E07 | Iodine related struma  Subclinical hypothyroid  Hypothyroidism  Atoxic struma  Thyrotoxicosis  Thyroiditis  Other thyroid disease |
| **Viral Hepatitis** | ICD code ever | B15  B16  B17  B18  B19 | Hepatitis A  Hepatitis B  Acute hepatitis C, D, E  Chronic hepatitis  Unspecified viral hepatitis |
