# Supplementary material for: The Association Between Number of Chronic Conditions and Benzodiazepine Prescribing in Region Stockholm, Sweden: A Total Population‐Based Cohort Study
Source: Health Sci Rep. 2026 Aug 9;9(8):e72970. doi: 10.1002/hsr2.72970 (PMC13454220; doi:10.1002/hsr2.72970)
Supplement: Supplementary file 3 [file HSR2-9-e72970-s003.docx]

Supplementary Material

# **Table 3.** Potentially inappropriate benzodiazepine prescribing before and during COVID-19 pandemic. Wash out analysis.

| **Number of chronic diseases** | Women, OR (99% CI) | | Men, OR (99% CI) | |
| --- | --- | --- | --- | --- |
|  | Before COVID-19 | During COVID-19 | Before COVID-19 | During COVID-19 |
| 2 | 3.81 (3.61-4.02) | 3.30 (3.30-3.31) | 2.54 (2.54-2.54) | 4.16 (3.82-4.51) |
| 3 | 5.87 (5.54-6.23) | 5.57 (5.56-5.57) | 4.44 (4.44-4.45) | 6.01 (5.47-6.60) |
| 4 | 8.41 (7.86-9.01) | 8.50 (8.49-8.51) | 8.34 (8.32-8.35) | 8.73 (7.86-9.71) |
| 5-9 | 23.98 (14.66-39.24) | 16.82 (16.82-16.86) | 25.93 (25.90-25.96) | 12.43 (11.21-13.77) |
| ≥10 | NA* | NA* | NA* | NA* |

Logistic regression model. The outcome measurement *Potentially inappropriate prescribing* was defined as ≥2 collected prescriptions of benzodiazepines during two years of time before, 20180101 to 20191231, and during COVID-19 pandemic, 20200101 to 20211231. Exposure was number of chronic diseases the four previous years to outcome measurement, 20140101 to 20171231 and 20160101 to 20191231 respectively. Reference was 0-1 chronic disease. The logistic regression model was adjusted for age as a continuous variable in years, and socioeconomic status as a categoric variable using Mosaic areas, representing neighborhoods with high (1), medium (2), and low (3) socioeconomic status(34,35). All individuals with benzodiazepines prescribed the last two years of exposure were excluded January 1, 2016, until December 31, 2017, and January 1, 2018, until December 31, 2019 respectively.

*Not available
