# Supplementary material for: The Association Between Number of Chronic Conditions and Benzodiazepine Prescribing in Region Stockholm, Sweden: A Total Population‐Based Cohort Study
Source: Health Sci Rep. 2026 Aug 9;9(8):e72970. doi: 10.1002/hsr2.72970 (PMC13454220; doi:10.1002/hsr2.72970)
Supplement: Supplementary file 4 [file HSR2-9-e72970-s004.docx]

Supplementary Material

# **Table 2a.** Baseline data of individuals with and without ≥2 collected prescriptions of benzodiazepines before COVID-19 pandemic. Wash out analysis.

|  | Women | | | Men | | |
| --- | --- | --- | --- | --- | --- | --- |
|  | ≥ 2 collected prescriptions of Benzodiazepines 2018-2019 | | | ≥ 2 collected prescriptions of Benzodiazepines 2018-2019 | | |
|  | Yes | No | Total | Yes | No | Total |
| **Total number (N) and percent of patients with or without a positive outcome measure** | N=19805  2.9% | N=657847  97.1% | N=677652  100% | N=10134  1.6% | N=619909  98.4% | N=630043  100% |
| **Number of chronic diagnoses** |  | | | | | |
| Mean number: | 2.6 (SD 1.8) | 1.0 (SD 1.3) | 1.0 (SD 1.3) | 2.5 (SD 2.0) | 0.9 (SD 1.3) | 0.9 (SD 1.4) |
| Categories: |  |  |  |  |  |  |
| 0-1 | 30.3% | 74.6% | 73.3% | 35.3% | 77.8% | 77.2% |
| 2 | 23.9% | 13.6% | 13.9% | 22.3% | 11.1% | 11.2% |
| 3 | 18.9% | 6.6% | 7.0% | 16.4% | 5.7% | 5.9% |
| 4 | 12.7% | 3.0% | 3.3% | 11.3% | 2.9% | 3.0% |
| 5-9 | 14.0% | 2.1% | 2.5% | 14.3% | 2.5% | 2.7% |
| ≥10 | 0.20% | 0.01% | 0.02% | 0.38% | 0.02% | 0.03% |
| **Age** |  |  |  |  |  |  |
| Mean age (in years) | 57.2 (SD 17.1) | 46.8 (SD 16.8) | 47.1 (SD 16.9) | 54.0 (SD 16.1) | 45.8 (SD 16.3) | 45.9 (SD 16.3) |
| 18-44 years | N=4986  1.6% | N=314954  98.4% | N=319940  100% | N=2941  1.0% | N=304670  99.0% | N=307611  100% |
| 45-64 years | N=7036  3.1% | N=223565  96.9% | N=230601  100% | N=4175  1.9 % | N=217500  98.1% | N=221675  100% |
| 65-79 years | N=6015  5.6% | N=101686  94.4% | N=107701  100% | N=2546  2.8% | N=87680  97.2% | N=90226  100% |
| 80+ years | N=1768  9.1% | N=17642  90.9% | N=19410  100% | N=472  4.5% | N=10059  95.5% | N=10531  100% |
| **Socioeconomic status**  Proportion of participants in mosaic group 1, 2, and 3^1^ (In percent) | 1: 28.4%  2: 36.3%  3: 34.7% | 1: 30.6%  2: 39.3%  3: 29.3 % | 1: 30.6%  2: 39.2%  3: 29.4% | 1: 30.5%  2: 35.2%  3: 33.0% | 1: 30.5%  2: 39.8%  3: 28.8% | 1: 30.5%  2: 39.7%  3: 28.9% |
| **Number of visits in primary care^2^** (Mean number (N) and (Standard deviation (SD))) | 4.5 (SD 5.2) | 2.2 (SD 3.2) | 2.3 (SD 3.3) | 3.7 (SD 4.9) | 1.6 (SD 3.0) | 1.6 (SD 3.0) |
| **Number of visits in secondary care^2^** (Mean number (N) and (Standard deviation (SD)) | 5.6 (SD 7.3) | 2.2 (SD 4.0) | 2.3(SD 4.1) | 5.0 (SD 8.1) | 1.5 (SD 3.9) | 1.6 (SD 4.0) |
| **Number of hospital admissions** (Mean number (N) and Standard deviation (SD))) | 0.5 (SD 1.5) | 0.1 (SD 0.6) | 0.2 (SD 0.6) | 0.5 (SD 1.6) | 0.1 (SD 0.6) | 0.1 (SD 0.6) |
| **Proportion of participants with ≥ 1 psychiatric diagnoses^3^** (In percent) | 73.5% | 26.7% | 28.1% | 70.6% | 17.2% | 18.1% |
| **Proportion of participants with polypharmacy^4^** (In percent) | 90.3% | 37.0% | 38.6% | 83.3% | 25.3% | 26.2% |
| **Proportion of collected prescriptions of Benzodiazepines prescribed in:**  *- Primary care (P)*  *- Secondary care (S)*  *-Both (B)*  (In percent) | P: 58.0%  S: 26.0 %  B: 16.1% |  |  | P: 56.5%  S: 28.3%  B: 15.3% |  |  |

Descriptive data analysis of all individuals with and without ≥2 collected prescriptions of benzodiazepines from January 1, 2018, until December 31, 2019. All individuals with benzodiazepines prescribed January 1, 2016, until December 31, 2017, were excluded.

*^1^Mosaic group 1 is defined as having high socioeconomic status, group 2 to have intermediate socioeconomic status, and group 3 to have low socioeconomic status.*

*^2^Defined as visits to a physician*

*^3^Phsyciatric diagnoses defined according to ICD-10.*

*^4^Participants with ≥ 5 collected prescriptions of medications.*

# **Table 2b.** Baseline data of individuals with and without ≥2 collected prescriptions of benzodiazepines before COVID-19 pandemic. Wash out analysis.

|  | Women | | | Men | | |
| --- | --- | --- | --- | --- | --- | --- |
|  | ≥ 2 collected prescriptions of Benzodiazepines 2018-2019 | | | ≥ 2 collected prescriptions of Benzodiazepines 2018-2019 | | |
|  | Yes | No | Total | Yes | No | Total |
| **Total number (N) and percent of patients with or without a positive outcome measure** | N=16973  2.4% | N=678211  97.6% | N=695184  100% | N=8586  1.3% | N=639533  98.7% | N=648119  100% |
| **Number of chronic diagnoses** |  | | | | | |
| Mean number: | 2.8 (SD 1.9) | 1.0 (SD 1.4) | 1.1 (SD 1.4) | 2.6 (SD 2.1) | 0.9 (SD 1.4) | 1.0 (SD 1.4) |
| Categories: |  |  |  |  |  |  |
| 0-1 | 27.2% | 72.8% | 71.7% | 33.8% | 76.1% | 75.5% |
| 2 | 22.1% | 14.0% | 14.2% | 20.7% | 11.4% | 11.5% |
| 3 | 19.8% | 7.1% | 7.4% | 16.2% | 6.2% | 6.3% |
| 4 | 17.1% | 2.7% | 3.6% | 12.4% | 3.3% | 3.4% |
| 5-9 | 17.1% | 2.7% | 3.0% | 16.3% | 3.1% | 3.2% |
| ≥10 | 0.4% | 0.02% | 0.03% | 0.5% | 0.03% | 0.04% |
| **Age** |  |  |  |  |  |  |
| Mean age (in years) | 57.5 (SD 17.1) | 47.1 (SD 16.8) | 47.3 (SD 16.9) | 54.5 (SD 16.3) | 46.1 (SD 16.3) | 46.2 (SD 16.3) |
| 18-44 years | N=4170  1.3% | N=321395  98.7% | N=325565  100% | N=2447  0.8% | N=311292  99.2% | N=313739  100% |
| 45-64 years | N=5975  2.5% | N=230722  97.5% | N=236697  100% | N=3487  1.5% | N=224830  98.5% | N=228317  100% |
| 65-79 years | N=5348  4.7% | N=108122  95.3% | N=113470  100% | N=2222  2.3% | N=92900  97.7% | N=95122  100% |
| 80+ years | N=1480  7.6% | N=17972  92.4% | N=19452  100% | N=430  3.9% | N=10511  96.1% | N=10941  100% |
| **Socioeconomic status**  Proportion of participants in mosaic group 1, 2, and 3^1^ (In percent) | 1: 27.8%  2: 37.0%  3: 34.9 % | 1: 30.6%  2: 39.7%  3: 29.5% | 1: 30.5%  2: 39.6%  3: 29.6% | 1: 29.9 %  2: 37.1%  3: 32.3% | 1: 30.4 %  2: 40.1%  3: 29.1% | 1: 30.4%  2: 40.1%  3: 29.1% |
| **Number of visits in primary care^2^** (Mean number (N) and (Standard deviation (SD))) | 4.2 (SD 4.7) | 2.1 (SD 2.9) | 2.1 (SD 3.0) | 3.4 (SD 4.3) | 1.5 (SD 2.6) | 1.5 (SD 2.6) |
| **Number of visits in secondary care^2^** (Mean number (N) and (Standard deviation (SD)) | 5.6 (SD 7.6) | 2.2 (SD 3.9) | 2.3 (SD 4.0) | 4.8 (SD 8.3) | 1.5 (SD 3.9) | 1.6 (SD 4.0) |
| **Number of hospital admissions** (Mean number (N) and Standard deviation (SD))) | 0.5 (SD 1.5) | 0.13 (SD 0.6) | 0.1 (SD 0.6) | 0.5 (SD 1.6) | 0.11 (SD 0.6) | 0.1 (SD 0.6) |
| **Proportion of participants with ≥ 1 psychiatric diagnoses^3^** (In percent) | 76.0% | 28.7% | 29.8% | 72.5% | 18.6% | 19.3% |
| **Proportion of participants with polypharmacy^4^** (In percent) | 91.4% | 37.6% | 38.9% | 85.0% | 25.9% | 26.7% |
| **Proportion of collected prescriptions of Benzodiazepines prescribed in:**  *- Primary care (P)*  *- Secondary care (S)*  *-Both (B)*  (In percent) | P: 58.8%  S: 26.9%  B: 14.2% |  |  | P:56.4 %  S: 29.7%  B: 13.9% |  |  |

Descriptive data analysis of all individuals with and without benzodiazepines ≥2 collected prescriptions from January 1, 2020, until December 31, 2021. All individuals with benzodiazepines prescribed January 1, 2018, until December 31, 2019, were excluded.

*^1^Mosaic group 1 is defined as having high socioeconomic status, group 2 to have intermediate socioeconomic status, and group 3 to have low socioeconomic status.*

*^2^Defined as visits to a physician.*

*^3^Phsyciatric diagnoses defined according to ICD-10.*

*^4^Participants with ≥ 5 collected prescriptions of medications.*
